# Heat Shock Transcriptional Responses in an MC-Producing Cyanobacterium (*Planktothrix agardhii*) and Its MC-Deficient Mutant under High Light Conditions

**DOI:** 10.1371/journal.pone.0073198

**Published:** 2013-09-04

**Authors:** Thi Du Chi Tran, Cecile Bernard, Myriam Ammar, Soraya Chaouch, Katia Comte

**Affiliations:** UMR Molécules de Communication et Adaptation des Microorganismes, Muséum national d’Histoire naturelle, Paris, France; University of New South Wales, Australia

## Abstract

Microcystins (MCs) are the most commonly-reported hepatotoxins produced by various cyanobacterial taxa in fresh waters to constitute a potential threat to human and animal health. The biological role of MCs in the producer organisms is not known, and it would be very useful to understand the driving force behind the toxin production. Recent studies have suggested that MCs may have a protective function in cells facing environmental stress. Following this starting premise, we speculate that under adverse conditions the expression of stress-related genes coding for Heat Shock Proteins (Hsp) might be different in an MC-producing strain and its MC-deficient mutant. We therefore used RT-qPCR to compare the expression of 13 *hsp* genes of an MC-producing strain of *Planktothrix agardhii* (CYA126/8) and its MC-deficient *ΔmcyD* mutant over different periods of exposure to high light stress (HL). Three reference genes (RGs) were selected from six candidates to normalize the RT-qPCR data. Of these three RGs (*rsh*, *rpoD,* and *gltA*), *gltA* is used here for the first time as an RG in prokaryotes. Under HL stress, five genes were found to be strongly up-regulated in both strains (*htpG*, *dnaK*, *hspA*, *groES,* and *groEL*). Unexpectedly, we found that the MC-producing wild type strain accumulated higher levels of *htpG* and *dnaK* transcripts in response to HL stress than the MC-deficient mutant. In addition, a significant increase in the *mcy*E transcript was detected in the mutant, suggesting that MCs are required under HL conditions. We discuss several possible roles of MCs in the response to HL stress through their possible involvement in the protective mechanisms of the cells.

## Introduction

Cyanobacterial bloom-forming species are a worldwide problem, because of the toxins they produce. The situation has become a cause of increased concern in recent decades as the frequency and intensity of bloom occurrence have increased, due in part to anthropic activities [1]. One of the diverse groups of cyanotoxins, that of the hepatotoxin microcystins (MCs) which includes up to 90 variants [2], is widespread and frequently reported. Microcystins [3] are becoming a real threat to human and animal health due to the contamination of freshwater [4]. However, the ecological significance and biological function of the MCs for the producer cells, which could elucidate the forces underlying toxin production, remain elusive. MCs are typically intracellular components, which are usually released into the environment after the cells die [5]. It has been shown that they are not essential for growth, but are probably involved in intracellular processes [6]. Various different hypotheses for the possible role of cyanotoxins have been proposed, these include a role as: grazer deterrent compounds [7] iron scavenging molecules [8], allelopathic compounds [9], growth regulators permitting successful adaptation [10], light harvesting and chromatic adaptation [11], and infochemicals [11]–[13]. However, the findings of the various studies are contradictory in many respects, and difficult to understand [14].

More recently, emerging investigations have suggested that MCs may have a protective role in the response to unfavorable conditions. Significantly greater growth rates have been observed in an MC-producing strain *Planktothrix agardhii*, than an MC-free strain; both strains had previously been collected from environmental samples (*i.e*. had different genomes) [15]. It has been also reported that transcription of the genes responsible for MCs production in *Microcystis* increased in response to strong illumination or iron starvation suggesting that MCs may play a protective role under various stressful conditions, including oxidative stress [11], [16]. To date, most of the data about the physiological roles of MCs has been reported by Neilan and colleagues in *Microcystis* [17], where they show that MCs production has a complex and deep effect on the proteome. Furthermore, MCs may be involved in the carbon-nitrogen metabolism, in redox control, in the perception of redox changes, and in providing protection against oxidative stress [17]–[19]. However, cyanobacterial responses to abiotic stresses are complex, and several mechanisms usually act in concert and synergistically to prevent cell damage and to re-establish cellular homeostasis [20]–[21]. The activities of heat shock proteins (hsps) constitute an important component of the cell’s response to stress. Depending on their size, Hsps are divided into 5 main classes: Hsp100, Hsp90, Hsp70, Hsp60 and small Hsp; representatives of each class are found in cyanobacteria [21]. Under normal conditions, Hsps play an important role in the folding, assembly, and trafficking of newly-synthesized polypeptides and in the degradation of denatured or aggregated proteins. Under stressful conditions, as a result of increased levels of aberrant proteins, the importance of Hsps increases, and this is usually reflected in their up-regulation [20]. However, much remains to be learned about the “*in vivo”* function of the Hsps proteins in cyanobacteria, as their biological role may extend to stress responses in general (known as HSRs for Heat shock responses) [22]–[23], and to multiple molecular cell defenses. Indeed, the protective effects of Hsps can be attributed to the network of the chaperone machinery, in which many Hsps play complementary and sometimes overlapping roles [20]. These major studies are initiated at the transcriptional level, using RT-qPCR (real-time quantitative polymerase chain reaction) analysis, which is one of the most powerful tools available for investigating quantitative differences in gene expression responses under experimental conditions [24]. However it is also a demanding tool, and calls for preliminary evaluation of the stability of a panel of reference genes (RGs) to provide accurate normalization of the gene expression analysis.

A few studies have identified the intracellular function of MCs in cyanobacteria against abiotic stressors at the transcriptomic level, and they involved the use of two strains sharing identical genomes, but with one gene engineered to inhibit MCs production. Unicellular *Microcystis* strains were investigated [19], but no study has yet been carried out of two identical clones of filamentous *Planktothrix agardhii* (differing solely by a single insertional mutation in one *mcy* gene), despite its hazardous impact on ecosystem functioning and the current increase of blooms in waterbodies. We selected *P. agardhii* for our study, due to its ecological preference for low light intensities [25], as HL (high light) could be expected to induce more explicit responses. We attempted to i) validate that the RGs were stable under our experimental conditions (*i.e.* control *vs* stressed conditions) in order to optimize RT-qPCR accuracy; ii) determine the gene expression profiles of 13 *hsps* and one *mcy* gene (*mcyE*, which is involved in the synthesis of Adda, and the incorporation of D-glu during MCs production); iii) determine the fold changes in the expression levels of the *hsps* and *mcyE* in the MC-producing strain (CYA126/8) and its MC-free mutant (*ΔmcyD)* when exposed to HL stress, using RT-qPCR analysis. To the best of our knowledge, this is the first study to report the transcriptional shift of a panel of 13 stress related genes (*hsps*) in MC-producing cells during a short period of exposure to stress (5–24 h).

## Materials and Methods

### Strains and Culture Conditions

Two strains of *P. agardhii* were used in this study. The MC-producing strain CYA126/8 (*i.e.* the wild type: WT) and its MC-deficient mutant (*ΔmcyD*) were kindly provided by Dr Kurmayer (University of Vienna). Both strains were monoclonal and not axenic and are maintained in the PMC (Paris Museum Collection, Paris). They shared an identical genome except that in the *ΔmcyD* mutant; a chloramphenicol cartridge was inserted into the *mcyD* gene to inhibit MC biosynthesis. We used LC/MS to confirm that CYA126/8 WT was an MC-producer, and that the mutant was free of MCs (Combes, unpublished data). Both cultures were maintained in Z8 liquid medium [26] at 20°C under white light (Osram white FM 11W/730 universal white) at 22±2 µmol.m^−2^.s^−1^ and with a light/dark cycle of 16/8. Cultures of mutant (*ΔmcyD*) cells were maintained under a constant selective pressure with chloramphenicol (1 µg/ml Z8 medium) to avoid potential wild type copies to grow (as the cyanobacteria may contain several genome copies), which may lead to the restoration of MC-producing cells. To avoid any effect that chloramphenicol may have had on physiological processes, two weeks before each experiment, the mutant culture was transferred to chloramphenicol-free Z8 medium.

### Experimental RT-qPCR Conditions

The cultures under optimal conditions (*i.e.* control) were obtained during the exponential growth phase under a continuous light intensity of 22 µmol m^−2^ s^−1^. For the HL treatment, the cultures in the exponential phase were shifted from control conditions to an intensity of 600 µmol m^−2^ s^−1^ during 24 h. For both conditions, the temperature was maintained at 20°C using a Binder phytotron. Experiments were performed using equivalent culture densities (OD_750 nm_ = 0.3) of both strains. Samples were taken at 0 h (control), and 1 h, 2 h, 5 h and 24 h after transferring to HL conditions, and were used for subsequent analysis. Two independent replicates were performed.

### RNA Extraction and cDNA Synthesis

For each sample, 40 ml of the culture suspension (OD_750 nm_ = 0.3) was centrifuged at 4°C, for 15 min, at 4000 rpm. Total RNA extraction was carried out using Trizol reagent (Invitrogen, USA) followed by purification using PureLink™ RNA Mini Kit (Invitrogen), according to the Manufacturer’s instructions. The pellet was mixed with 3 ml of Trizol, and then immediately frozen in liquid nitrogen and conserved at −80°C until extraction. Phase separation was obtained by adding 600 µl of chloroform to the cell lysate and shaking vigorously by hand for 15 seconds (8 times), storing at room temperature for 5 minutes, and then centrifuging at 12 000 g for 15 minutes at 4°C. RNA purification was performed using PureLink™ RNA Mini Kit (Invitrogen). Purified RNA, previously treated with a DNA-free Kit (Ambion), was quantified using a NanoDrop 2000 Spectrophotometer (Thermo Scientific); and its integrity was checked on 1.5% agarose gel (data not shown). Genomic DNA contamination was checked by PCR on a total RNA template using primers targeting the citrate synthetase sequence (data not shown).

The A_260_/A_280_ ratio of the RNA samples was 2.089±0.017 (mean ± SD), indicating the absence of protein and the purity of all the total RNA samples required for an accurate qRT-PCR analysis. First-strand cDNA was synthesized from 0.8 µg total RNA using SuperScript III First-Strand Synthesis SuperMix (Invitrogen, Carlsbad, USA), with 1 µl of random hexamers in a 20-µl reaction mixture, according to the Manufacturer’s instructions. cDNA samples were stored at −20°C.

### Genes Investigated in the Study

Two series of genes were used in this study (Table 1). As the relevance of RT-qPCR analysis greatly depends on transcript normalization with stably-expressed reference genes (RGs), 6 candidates were selected from different functional classes. Four conventional candidate RGs were tested: *16S rRNA* [27], *rpoD* [28], *GAPDH* [27], *rsh* [27], plus two RGs genes that had never so far been tested in prokaryotes: *gltA* and *rpsL*. The sequence of *16S rRNA* is available in a database (GeneBank FJ184435.1), but the other 5 RGs candidates were all isolated in this study.

**Table 1:** Information about the genes investigated in this study.

Gene	Name	Description/function	Accession number
*Rsh* *	(p)ppGpp synthase/hydrolase	Control of metabolism of (p)ppGpp thereby involved in responses to nutritionaldeprivation	KF275118
*rpoD* *	RNA polymerase sigma factor	Primary RNA polymerase sigma factor	KF275120
*gltA* *	Citrate synthase	Citric acid cycle	KF275124
*GAPDH* *	Glyceraldehyde 3-phosphatedehydrogenase	Glycolysis	KF275123
*rpsL* *	30S ribosomal protein S12	Structural constituent of ribosome	KF275122
*16S rRNA* *	16S ribosomal RNA	Structural constituent of ribosome, acting as scaffold defining the positions ofribosomal proteins	FJ184435.1
*hspA* **	Small heat shock protein	Prevent irreversible protein aggregation during stress	KF294790
*hslO* **	33 kDa heat shock protein	Chaperon holdase, functioning as a first line of defense during oxidative stressconditions that cause protein unfolding.	KF294782
*hsp40* **	40 kDa heat shock protein	Co-chaperone of Hsp70, regulating complex formation between Hsp70 and client proteins.	KF294789
*grpE* **	Nucleotide exchange factorfor DnaK	Stimulate the release of ADP from Hsp70, fostering substrate dissociation,thereby ‘recycling’ Hsp70 molecule.	KF294788
*dnaK* **	70 kDa heat shock protein	Help the folding of nascent proteins under normal conditions, prevent the aggregationof unfolding proteins and assist in refold aggregated proteins under stress conditions.	KF294783
*hsp70(1)* **			KF294784
*hsp70(2)* **			KF294785
*hsp70(3)* **			KF294786
*hsp70(4)* **			KF294787
*clpC* **	100 kDa heat shock protein	Regulatory ATPase/chaperone subunit of Clp protease, involved in the efficientdegradation of irreversibly damaged proteins.	KF275115
*htpG* **	90 kDa heat shock protein	Recognize and bind non-native proteins to prevent their nonspecific aggregation	KF275116
*groEL* **	60 kDa heat shock protein	Bind to partially folded/unfolded protein and enable them to fold in a protectedenvironment where they do not interact with any other proteins.	KF275121
*groES* **	10 kDa heat shock protein	Co-chaperone of GroEL	KF275119

* Reference gene candidates;

** Target genes.

The second series were the target genes. They included nine *hsp* genes: *hspA*, *hslO*, *hsp40*, *grpE, dnaK, hsp70 (1), hsp70(2), hsp70(3),* and *hsp70(4),* which had previously been sequenced, and were kindly provided by Dr Quiblier (MNHN, Paris) plus four other genes: *clpC* (*hsp100*), *htpG* (*hsp90*), *groEL* (*hsp60*) and *groES* (*hsp10*)), which were isolated in this study. The full-length sequences of the genes obtained here are available in the EMBL database under the following accession numbers: KF275115 to KF275124, and KF294782 to KF294790. We also included *mcyE,* which is involved in Adda synthesis and the incorporation of D-Glu in the biosynthesis of MCs [29] as a target gene.

### Primer Design and qPCR Conditions

All primer sets except that of *mcyE* (Table 2) were manually designed and then analyzed using NetPrimer algorithm, PREMIER Biosoft International (http://www.premierbiosoft.com/netprimer/). Furthermore, all primer pairs were checked for specificity using Primer-BLAST [30]. For *mcyE*, the primers mcyE-plaR3 [31] and mcyE-F2 [32] were used.

**Table 2:** Real-time PCR primers used in this study.

Name	Primer sequence (5′ –3′)		Amplicon size (bp)
	Forward	Reverse	
*HspA*	GCGATGTCCCTCTTTCCTCC	CCTTTTTCTTCGGTTTGGTTG	167
*HslO*	CCACATCCAGAGTCAATATCCG	CCATAACCAACATCTCGCACC	202
*Hsp40*	ACCTGCGTTTAGAGTTCAAAGAAG	CGGACAAACGGAAACCTGAG	206
*GrpE*	GCGAATAATCCTGATGAACTAACG	CGCTTGATTCTATTTCCTGACC	181
*DnaK*	GAACGCATTGAACGCAAAAAC	GCTTGTTGTAAATCCGTAGTTAGGG	191
*Hsp70(1)*	CTGCTAAACGGGGTATTCCTC	CATCTTCATCGGCATAAACTTCTG	189
*Hsp70(2)*	CATTGGCATAGACTTAGGGACAAC	GTATTTTCCGCATTGGTAACGAC	183
*Hsp70(3)*	CCCGTTGTGATTGCTAACTCTG	GTGTAGGGAACCCGTTTTGAG	200
*Hsp70(4)*	GTAACGGCAGAGGATAACACCC	CCCCTAACCAAACGGAAAGAC	259
*ClpC*	GTTTCCCGTGCCATTCGTC	GTTGTCCGCCTTCGTTGTATC	247
*HtpG*	GAACGCAATAAAGAACGCCAC	GTCATCTAGTTCCGCATCCACC	201
*GroEL*	GCTCAAGTCGGTTCTATCTCTGC	CTTCCATCCGTTCGGTATCG	205
*GroES*	CTGTATCTCTAAGCGTATCAACCG	CATCATTGCGTTTGCCAGG	168
*Rsh*	CCTCACCTACCTTCCTATTCTCAAC	CGAATCTTTCTCCCTCCACG	208
*RpoD*	GACTCGCAACCCTTCCACTG	CTTTGTTCTCGTCATCTTCCTCC	168
*GltA*	CCACCAAAGATGAGTTAGCAGAC	GGATTATCTAAAGCCCGACGAG	175
*GAPDH*	GAAAGGGTGAAGGCGTGG	GTGGGTTGTGGTCATTGTGC	173
*RpsL*	GCTAACCTCTGGCTATGAAGTGAC	CTCCCGCCGTATCTAATGTTC	152
*16S rRNA*	GGAGTACGCACGCAAGTGTG	GATGGCAACTAACGACGAGG	246
*mcyE*	GAAATTTGTGTAGAAGGTGC	CTCAATCTGAGGATAACGAT	250

The specificity of the primer sets were tested by Real-time PCR using cDNA of *P. agardhii* CYA 128/6 and confirmed by melting curve (Fig. S1) and gel electrophoresis (Fig. S2). The identities of all PCR products were further confirmed by TA cloning using pGEM-T Easy vector (Promega, WI, USA) and subsequent sequencing.

The melting curve analysis indicated that all the primer pairs produced a single peak (Fig.S1), and only one band of the expected size was obtained on 2.5% agarose gel electrophoresis, which confirmed the specificity of all the primer pairs (Fig. S2).

Real time PCR was performed on a LightCycler 2.0 (Roche) using 32-capillary carousel combined with the LightCycler FastStart DNA Master SYBR Green I (Roche). Each 20-µl capillary contained a total volume reaction of 10 µl including: 1 µl of ready-to-use hot start PCR reaction mix; 1 µl of primer mix (Table S1); 4 µl of 1∶64 diluted cDNA sample; and 4 µl of MgCl_2_. Each run included a non-template control (NTC). Real-time amplification reactions for each gene of the WT and mutant strains from one biological replicate were performed in a technical duplicate and in the same PCR run. The cycling conditions were: 1 cycle at 95°C for 10 min, followed by 40 cycles at 95°C for 10 s, 62–65°C (depending on the target – Table S1) for 4 s, and 72°C for 10 s. In this study, the C_T_ was automatically identified using the “Second Derivative Maximum Method” [33]. At the end of the amplification, the melting temperature of the product was also determined using the melting curve program: 65–95°C, with a heating rate of 0.1°C per s and continuous fluorescence measurement.

### Expression Stability of the Candidate Reference gene and Data Analysis

In order to determine the true gene-specific variation, at least one stably-expressed RG is required to normalize the expression level of the target genes. The expression levels of the six candidate RGs were determined by RT-qPCR under the same experimental conditions as for the target genes (*i.e*. control condition+HL stress).

To identify the genes most stably expressed during HL treatment, the C_T_ values of these six candidate RGs were analyzed using three different mathematical algorithms: geNorm [34], Normfinder [35] and BestKeeper [36]. In brief, GeNorm is the one most commonly used in the literature and it relies on the transformation of raw C_T_ values (using the delta-C_T_ method). The gene expression stability measure (M) for a candidate RG is computed by averaging pairwise variations of that gene *versus* all the other candidates tested. A decrease in the M value reflects an increase in expression stability. NormFinder is a model-based algorithm used to identify the optimum RGs from a group of candidates. This algorithm required the transformation of C_T_ values to linear scale expression quantities. The genes with the lowest stability values have the most stable expression. BestKeeper uses the raw C_T_ as the input for calculation. A Pearson correlation coefficient was calculated for each candidate pair as well as the probability that the correlation was significant. An index value was calculated as the geometric mean of the C_T_ values of all highly-correlated candidate RGs. Stable RGs show a strong correlation with the BestKeeper index.

The gene-specific PCR efficiency was determined for each pair of primers using a 5-fold serial dilution of cDNA as template. The standard curve was obtained by plotting C_T_ values against a logarithm of serial dilutions of the target nucleic acid. The efficiency of the reaction (E) was calculated from the slope value of a standard curve, as follows: E = 10^(−1/sl^°^pe)^ –1.

The relative quantity of each gene (Q), which was used in geNorm and NormFinder, was calculated as: Q = E**^(min C^_T_** ^− **sample C**^ **_T_^)^** where Q = sample quantity relative to the sample with the highest expression; E = amplification efficiency; min C_T_ = lowest C_T_ value = C_T_ value of the sample with the highest expression.

To calculate the normalized relative gene expression levels, data were analyzed using Relative Expression Software Tool (REST) (http://gene-quantification.com/rest.html) [37].

Data of transcript expression levels were analyzed using one-way analysis of variance (ANOVA) at a confidence level of *p*<0.05; followed by Tukey’s test on GraphPad Prism 5.0 software.

## Results

### Selection and Validation of Reference genes for RT-qPCR Normalization

**Figure 1:**
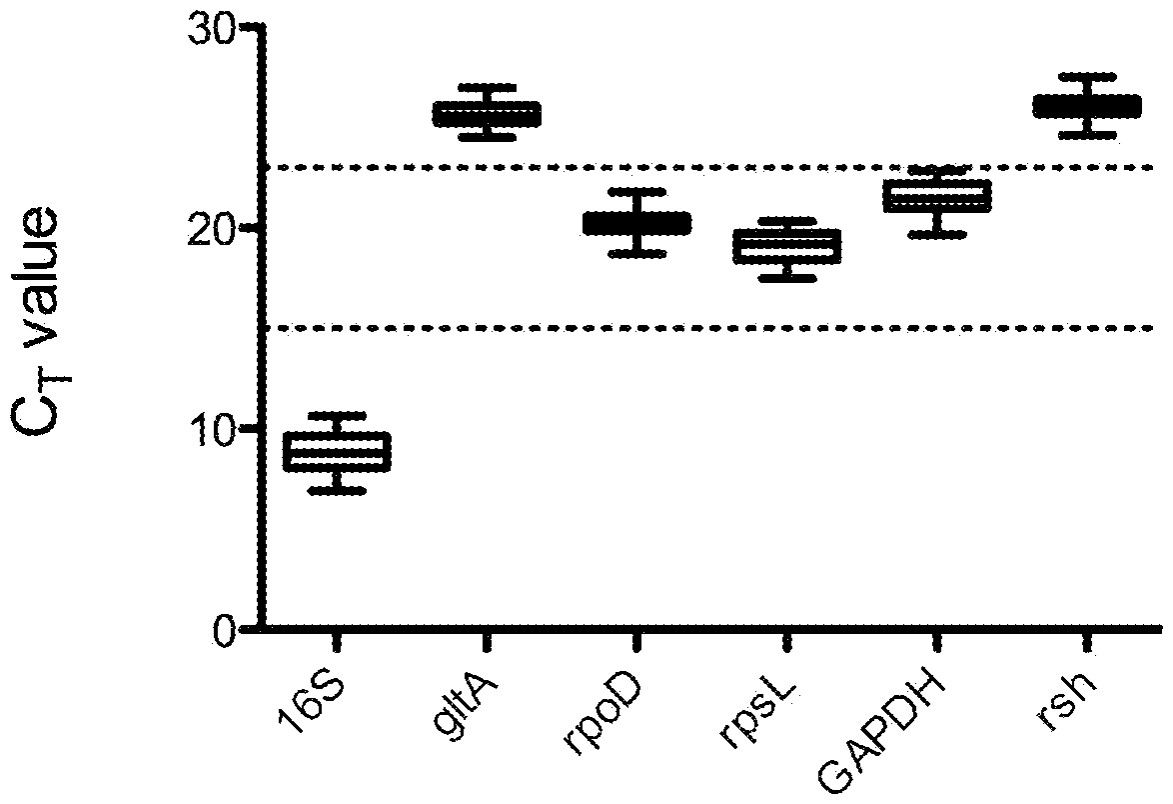
Real-time PCR C_T_ values in the samples collected. The distribution of the expression levels of candidate reference genes is shown by the median (lines), the lower and upper quartiles (boxes), and ranges (whiskers) (n = 20). The genes were divided into three groups by the arbitrary lines at C_T_ 15 and 23, on the basis of their different expression levels.

In order to compare the expression levels of *hsp* target genes in the WT and *ΔmcyD* mutant of *P. agardhii* CYA 126/8 under HL conditions, we normalized all the samples using the same RGs. The C_T_ values were obtained for each candidate gene across all the samples (Fig. 1) and revealed the differences in transcript levels. The *16S rRNA* gene gave the lowest C_T_ (8.81), corresponding to the highest expression level, whereas *gltA* and *rsh* showed the lowest expression levels, with mean C_T_ values of 25.66 and 25.99, respectively (Fig. 1). The expression stability of the candidate RGs were analyzed using geNorm, NormFinder and BestKeeper, which provided complementary measures of the cDNA samples. Both geNorm and NormFinder classified *16S rRNA* as the least stable gene (Table 3), regardless of the data series used (combined or single WT and mutant).The transcript level of this candidate was also much higher than that of the others (Fig. 1). Because it is crucial to use RGs with ranges of expression similar to those of the target genes in the samples for analysis [38], we excluded the *16S rRNA* gene from the BestKeeper analysis. The three most stable genes identified by all three programs were similar, especially for the first two in all the data sets (Table 3). *Rsh*, *rpoD* and *gltA* were identified as the best performing genes, whereas *rpsL* and *16S rRNA* were always identified as the least stable genes. The optimum number of RGs required for an accurate normalization was provided by the pairwise variation (V_n/n+1_) calculation using the GeNorm program. The closest value to 0.15 (the cut-off value for validation - [35]) in our analyses, was found for V_2/3_ (0.12–0.14 according to data series); the value for V_3/4_ was even lower (0.09), indicating that three reference genes were the optimal number for accurately normalized gene expression. Consequently, we validated the 3 most stable reference genes (*rsh, rpoD* and *gltA)* for the normalization of all RT-qPCR data.

**Table 3:** Ranking of candidate reference genes by three different algorithms.

Gene name	Ranking order												
	*geNorm*				*NormFinder*			*BestKeeper*					
	Combined	WT	M	No group		2-group			Combined		WT	M	
*rsh*	**1/2**	**1/2**	**1/2**		**1**		**1**	**1**		**2**			**1**
*rpoD*	**1/2**	**1/2**	**3**		**2**		**3**	**2**		**1**			**3**
*gltA*	**3**	**3**	**1/2**		**4/5**		**2**	**3**		**4**			**2**
*GAPDH*	**4**	**4**	**4**		**3**		**4**	**4**		**3**			**4**
*rpsL*	**5**	**5**	**5**		**4/5**		**5**	**5**		**5**			**5**
*16S rRNA*	**6**	**6**	**6**		**6**		**6**						

GeNorm (75), NormFinder (2) and BestKeeper (51) used to identify the most stably expressed genes in control and HL conditions. Wild type (WT) and mutant (M) strains of *Planktothrix agardhii*.

### Expression Levels of hsp genes of *P. agardhii* under Control Condition

**Figure 2:**
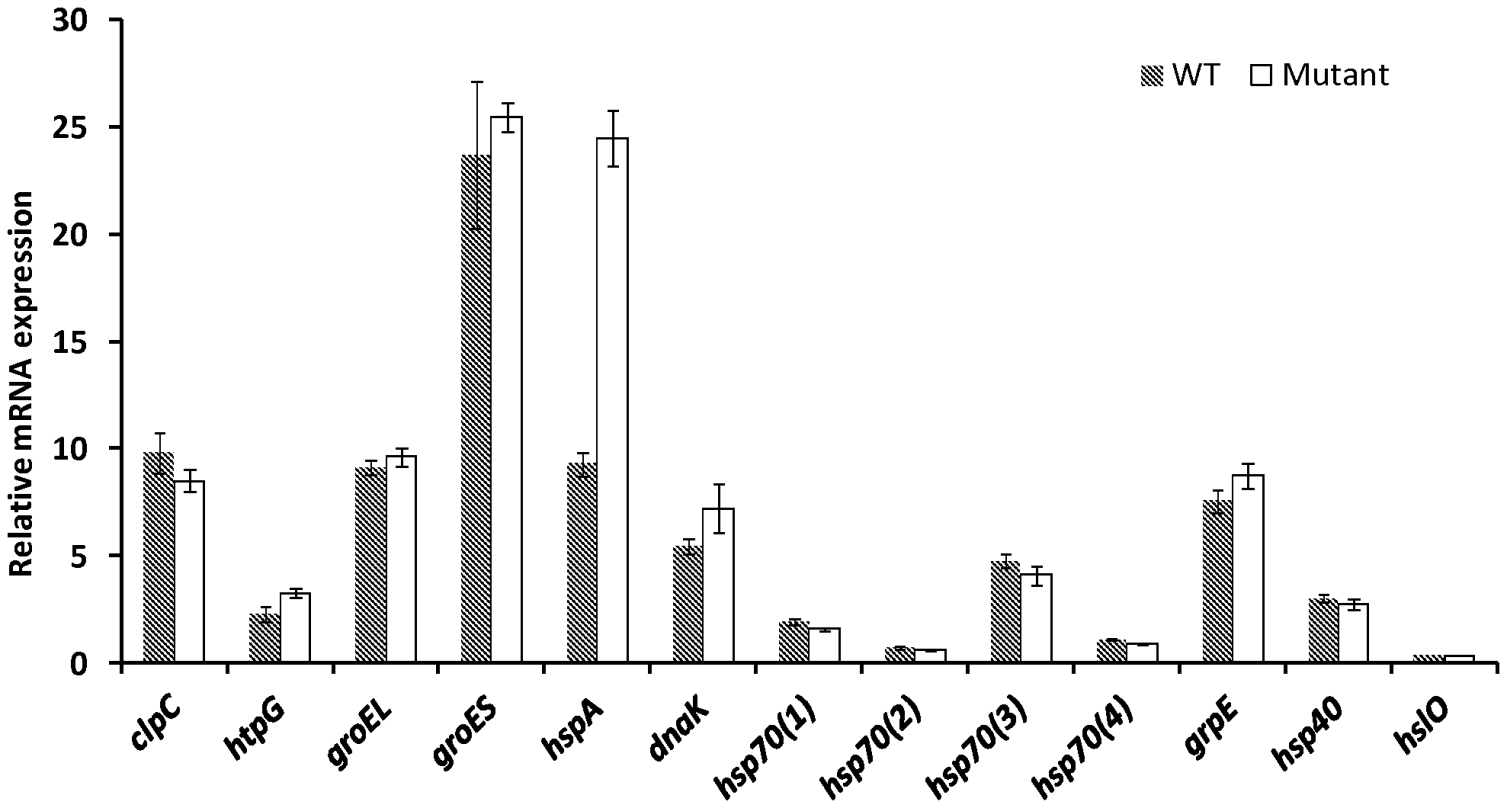
Relative mRNA expression levels of *P. agardhii hsp* genes under optimal conditions. Normalization against three references genes: *rsh*, *rpoD* and *gltA*. Error bars correspond to the standard deviation, including two technical replicates for two independent biological samples. Asterisks indicate a significant difference in the expression levels of the WT and the mutant strain; ***: *p*<0.001.

The expression level of the 13 target *hsp* genes (Fig. 2) was determined under control conditions *versus* the geometric mean of the transcription levels of the three selected reference genes. They could be divided into four groups (with no significant differences within mRNA abundance (p>0.05)) listed in decreasing order of mRNA abundance: (i) *groES* (WT and *ΔmcyD*) and *hspA* (*ΔmcyD* strain); (ii) *groEL*, *clpC*, *grpE* (WT and *ΔmcyD*) and *hspA* (WT); (iii) *htpG*, *dnaK*, *hsp70(3), hsp40* (WT and *ΔmcyD*), and (iv) *hsp70*(1), *hsp70*(2), *hsp70*(4), *hslO* (WT and *ΔmcyD*).

Under control conditions, the *groES* gene was the one most highly expressed in both the WT and *ΔmcyD* (23.72±3.45 and 25.47±0.7, respectively). Except for *hspA*, no significant difference was found under control conditions between the WT and *ΔmcyD* (p>0.05). The *hspA* expression level was significantly higher (p<0.001) in *ΔmcyD* than in WT (24.5±1.26 and 9.27±0.52, respectively).

### Effects of HL on the Expression Levels of hsp genes in *P. agardhii* WT and ΔmcyD Mutant

**Figure 3:**
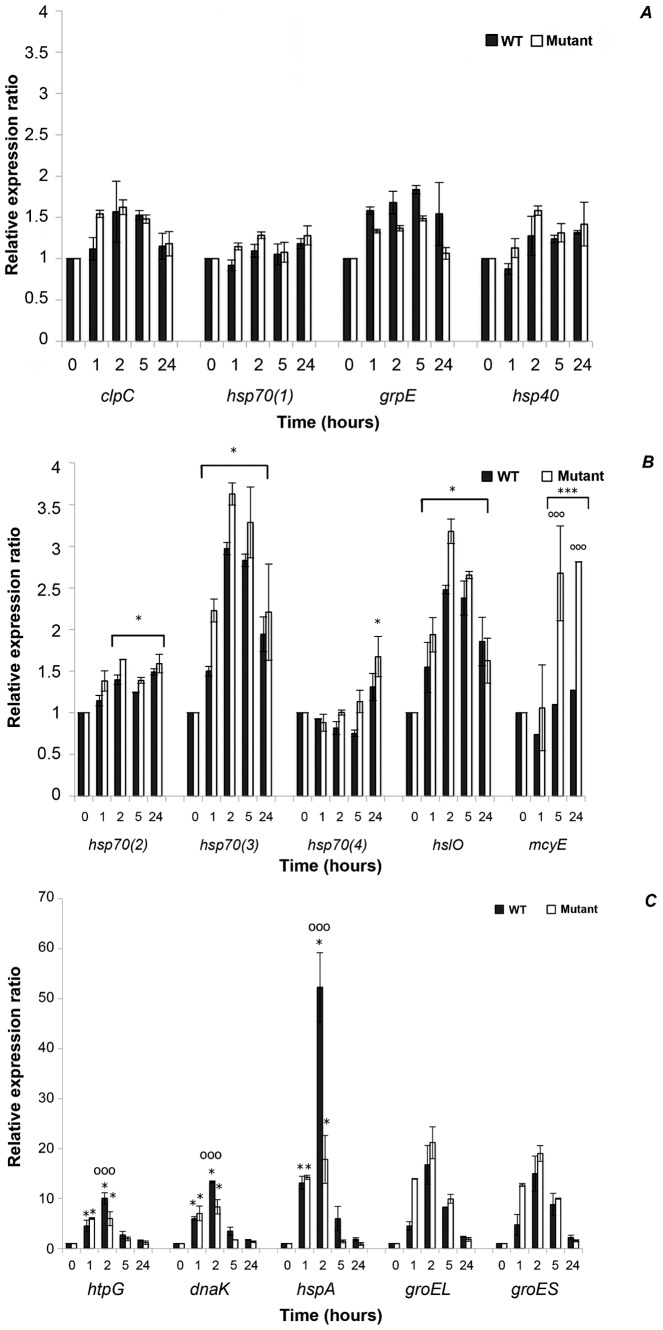
Relative expression levels of the *hsp* genes and *mcyE* gene of *P*. *agardhii* from control (T_0_) to high light stress (1 h to 24 h). Relative mRNA expression of *hsp* genes was normalized against three RGs: *rsh*, *rpoD* and *gltA*. (A): unchanged expression; (B): <4 fold up-regulated; (C): >4 fold up-regulated. Error bars correspond to the standard deviation, including two technical replicates for two independent biological samples. An asterisk indicates a significant difference *versus* control (T_0_) *: *p*<0.05. Circles indicate a significant difference in the expression level between the WT and the mutant strains; °°: *p*<0.01; °°°: *p*<0.001.

The transcriptional response of 13 *hsp* genes of *P. agardhii* (WT and *ΔmcyD*) was compared at different times (0, 1 h, 2 h, 5 h and 24 h) under HL conditions (Fig. 3 and Table 4). Based on the relative expression levels (shown as the fold change in gene expression *versus* control at time T_0_), three different expression profiles were obtained: (i) *clp*C, *hsp*70(1), *grp*E and *hsp*40 (Fig. 3.A) showed no significant difference in expression level (*p*>0.05) under control and HL conditions*;* (ii) *hsp70(2)*, *hsp70(3)*, *hsp70(4)* and *hslO* (Fig. 3.B) showed a slight (<4 fold) but significant change (p<0.05) in expression levels under HL and (iii) *htpG*, *dnaK*, *hspA, groEL* and *groES* (Fig. 3.C) showed a strong (>4 fold) and significant (*p*<0.05) increase in expression level under HL conditions.

**Table 4:** Relative fold change of transcript of the genes obtained by qRT-PCR.

	Gene	WT	M	Statistical difference between WT and Mutant
Group 1	*clpC*	1.57±0.37	1.62±0.09	NS
	*hsp70(1)*	1.1±0.08	1.28±0.04	NS
	*grpE*	1.68±0.14	1.37±0.03	NS
	*hsp40*	1.28±0.24	1.58±0.06	NS.
Group 2	*hsp70(2)*	1.40±0.06	1.64±0.001	*
	*hsp70(3)*	2.97±0.07	3.63±0.13	NS
	*hsp70(4)*	0.82±0.08	1.00±0.03	NS
	*hslO*	2.48±0.05	3.18±0.15	NS
	*mcyE*	1.1±0.13	2.7±0.57	***
Group 3	*htpG*	10.06±1.09	5.98±1.40	***
	*dnaK*	13.34±0.22	8.34±1.44	**
	*hspA*	52.31±6.90	17.82±4.80	***
	*groEL*	16.77±3.86	21.19±3.21	NS
	*groES*	14.98±3.53	19.02±1.59	NS

In the WT and mutant (M) strains of *P. agardhii* after exposure to HL stress for 2 h (for *hsp* genes) and 5 h (for *mcyE*). Data normalization was done using the three most stable RGs (*rsh*, *rpoD* and *gltA*). Group 1: genes which showed no significant change under HL conditions; Group 2: genes which were slightly up-regulated (<4 fold) under HL conditions; and Group 3: genes which were strongly up-regulated (>4 fold) under HL conditions. Values are reported as mean ± SD; NS: *p*>0.05;

* *p*<0.05;

** *p*<0.01;

*** *p*<0.001.

The expression of the following genes was significantly higher under HL than under control conditions (T_0_) (Fig. 3.B): *Hsp70(3)* and *hslO* showed a significant increase at 1 h, with a peak after 2 h under HL (≈3.5 fold for both strains) (*p*<0.05). *Hsp70(4)* exhibited a significant (*p*<0.01) increase after 24 hours for both the WT and the *ΔmcyD* (1.31±0.16 and 1.67±0.24, respectively). For *hsp70(2),* a slight but significant increase (*p*<0.05) was observed in the WT and the mutant after 2 hours under HL (1.38±0.12 and 1.64±0.001, respectively) (*p*<0.05).

Five genes were strongly up-regulated under the HL conditions (Fig. 3.C) and they displayed a similar expression pattern: a significant increase of expression level (*p*<0.001) at 1 hour, a peak reached after 1 to 2 hours under HL, and then a decrease to the background level. The expression profiles of *htpG, dnaK,* and *hspA* were quite similar in both strains. Interestingly, quantitatively significant differences were found between the WT and the *ΔmcyD* for these three genes (Fig. 3.C). In the WT, the expressions of *htpG, dnaK,* and *hspA* were up-regulated to a greater extent after 2 h than in *ΔmcyD* (Fig. 3.C). The fold change in *dnaK* expression induced by HL was 13.34 in the WT versus 8.34 in the *ΔmcyD* (*p*<0.01). For *htpG*, the expression fold change reached its maximum of 10.06±1.08 after 2 h of exposure to HL in the WT, which was significantly higher (*p*<0.001) than that in the mutant (5.98±1.39).

The relative expression of *hspA* after 2 h under HL was 52.31±6.9 in the WT strain compared to 17.82±4.8 in the *ΔmcyD* (*p*<0.001). However, it is noteworthy that under control conditions, the level of *hspA* in WT was significantly lower than in the Δ*mcy*D strain (9.27±0.52 and 24.5±1.26, respectively) (Fig. 2). Therefore, even though the maximum level of change in WT reached 52.31±6.9 fold *versus* 17.82±4.8 fold in the mutant, there was no significant difference between the amounts of *hspA* mRNA under HL in the two strains (*p*>0.05).

For *groEL* and *groES* genes, in both strains, the mRNA reached the greatest level 2 hours after being exposed to HL stress and then decreased (Fig. 3.C). However, the pattern of change differs in timing with a significant increase (*p*<0.001) right after 1 h in the mutant, in contrast to the unchanged value in the WT (Fig. 3.C). The maximum level was reached after 2 h in both strains. For *groEL* and *groES* expression in HL, no significant differences were observed between the maximum levels of the WT and *ΔmcyD*.

### Effects of HL on Expression Levels of the mcyE gene in *P. agardhii* WT and ΔmcyD Mutant

The transcription level of *mcy*E of the WT strain was not affected by HL during the test period (0–24 h) (Fig. 3.B). The main finding was that the insertion of the Cm^R^ gene into *mcyD* did not disrupt the expression of another *mcy* gene (*mcyE*) in *P. agardhii*. Under HL, a significant increase in the abundance of *mcy*E mRNA was observed in the mutant (2.7 fold 5 h after the shift from control conditions to HL) (*p*<0.001). This mRNA level remained high till 24 h.

## Discussion

There is a general consensus that the selection of suitable reference genes for normalization of the target genes is a prerequisite for RT-qPCR [24]. In this study, six candidate genes were chosen from independent pathways to avoid possible effects due to co-regulation. Four genes had previously been used as conventional RGs in prokaryotes (*16SrRNA, rpoD, GAPDH, rsh*) [27]–[28]. To the best of our knowledge, the other two candidate genes (*gltA, rpsL*) had never been tested in prokaryotes. A combination of three computational programs (GeNorm, NormFinder and BestKeeper) was used to provide accurate validation of the most stable genes for normalization [39]. The same three genes (*rsh*, *rpoD* and *gltA*) were identified as being the most stable genes, despite some slight differences in ranking order (Table 3). Since these three algorithms rely on different mathematical approaches to calculate stability (see Mat & Meth), these minor differences between their outputs were not unexpected [36], [40]. Such discrepancies have been reported in several studies as minor changes in gene stability rankings [41]. According to the geNorm analysis, V2/3 was below 0.15, indicating that the minimum number of RGs required for reliable normalization in this study would be two. However, it should be pointed out that using additional genes is usually an option [40]–[41], and using the three best RGs is a valid normalization strategy in most cases [35]. Moreover, in our study, including the third most stable candidate gene gave a significantly lower V-value. We therefore decided to use the three most stable genes as RGs for normalization of the RT-qPCR data. These included *gltA*, which will be listed as a newly-recommended RG for further RT-qPCR data normalization, because validation of the suitability of RGs for normalization in prokaryotes is still lacking [42].

The most unstable genes were also the same in all the sample sets (Table 3). We showed here that *16SrRNA* and *GAPDH*, two RGs used in previous studies [28], [43] were not appropriate for normalization under our experimental conditions for the two *Planktothrix* strains we used. The suitability of *16SrRNA* gene for use as an RG is currently disputed [44] due to its high abundance compared to target gene transcripts (resulting in too large a difference in C_T_ values), which can bias interpretations [17], [35]. This once more showed that appropriate RGs can be very different in different organisms as well as under different experimental conditions and the more commonly used RGs are not necessarily always suitable. Each RG needs to be validated under the same experimental set-up as the target gene. The validation of three RGs that are stably expressed under our experimental settings allowed us to evaluate the relative expression levels of 13 *hsp* genes of the MC-producing strain and of its MC-deficient mutant under optimum and HL conditions over a period of 24 h.

Under control conditions, all the *hsp* genes were found to be constitutively expressed. Among them, *groES* showed the highest expression level, which was at least double that of the others (Fig. 2). This was not surprising, as most Hsps are constantly produced to assist the proper folding of nascent proteins, and to prevent protein aggregation throughout the lifetime of cells [21], [45]. Furthermore, *groES* is a part of the *groESL* operon, a major chaperone system in bacteria, which plays an important role in the conformational homeostasis of cell proteins [38]. However, under control conditions, a higher expression level (about 2.5-fold greater) of a small *hsp* (*hspA*) was observed in the mutant strain than in the WT strain (Fig. 2), something that we cannot explain.

Under HL stress, among the 13 genes under investigation, only five *hsp*s were strongly up-regulated in both strains, with a 6 to 52-fold change relative to control conditions, four genes were slightly up-regulated, and the other four were unchanged (Fig. 3, Table 4). It has been observed that the Hsps stress response varies considerably in some organisms depending on the species, Hsp family, developmental stage, and stressor [46]–[47]. Diverse Hsp isotypes in different species may have different roles and modes of action. Therefore, the true significance and role of Hsps in difference species must be confirmed using methods appropriate for each species.

However, it had previously been reported that HL stress may induce changes in gene transcription within as little as 15 minutes, and the expression level may also return to the basal level very quickly [48]. In this study, over our experimental time course (1–24 h) the five genes were all strongly up-regulated and displayed a similar kinetic pattern. The expression level increased 1 h after the shift to high light; it then reached its maximum level after 1 to 2 h, and thereafter declined (Fig. 3). This pattern of change is typical of how gene expression usually responds to stress [48]. These changes are usually transient and, even with persistent stress, gene expression fairly soon reaches a new homeostasis, in which the physiology of the cell has adjusted to new conditions [48]–[50].

The pronounced inductions of the five *hsp* genes (*htpG, dnaK, hspA, groEL, groES*) are consistent with those reported for *Synechocystis* PCC6803, where *htpG, dnaK2, groESL,* and *hspA* were also conspicuously up-regulated in response to a shift from low light to HL [48], [51]. This may suggest that these genes play a significant physiological role in protecting cells against this specific abiotic stress, regardless of the cell’s ability to produce MCs.

The most important finding of our study was detecting some transcriptional differences between the WT and its MC-deficient mutant. Indeed, two *hsps* genes (*htpG* and *dnaK*) were ≈ 1.7-fold more highly expressed under HL stress in WT than in *ΔmcyD* (Table 4 and Fig. 3). HtpG had previously been reported to play a role in the ability of cyanobacteria to tolerate various stresses [52]–[54], including providing effective protection against the oxidative stress caused by HL in unicellular cyanobacteria [50]. Some studies have suggested that HtpG is involved in regulating the biosynthesis of tetrapyrrole [55] and that it interacts with the linker polypeptides of phycobilisome in cyanobacteria to prevent their thermal aggregation [56]. These activities may endow HtpG with an effective photoprotective role in response to HL stress.

For the *dnaK* gene, it has been shown that cyanobacteria contain multiple *dnaK* homologs, the expressions of which are differently regulated [57]. However, the alignment of our sequence (Accession no.: KF294788) revealed the greatest similarity to *dnaK2* of *Synechocystis* PCC6803 (91% similarity- data not shown). *dnaK2* has been reported to be induced by various abiotic stresses [50], [58]–[59], including HL [50], although its essential function is still elusive [60].

Even if their multiple functions are still unclear, one would expect that higher expression levels of *dnaK* and *htpG* may contribute to better protection of macromolecular complexes, such as the photosynthetic apparatus, and thus enable the WT strain to tolerate HL better.

Finally, an unexpected finding was about the expression profile of the *mcyE* in both strains when they were transferred from control conditions to HL stress (Fig. 3.B, Table 4). In the WT strain, stable expression of the *mcyE* gene was observed after the transition from control to HL stress conditions for a short period of time (0–24 h), in contrast to some previous findings in *Microcystis* strains. An up-regulation of *mcyB* and *mcyD* was found as a result of HL intensities [11]. Using RT-qPCR we showed that the expression level remained constant, which was corroborated by constant MCs production by the *P. agardhii* WT cells during the first 24 h (450 ng eq. MC-LR per mL of culture normalized to OD_750 nm_ = 1, unpublished data). The *mcy* operon seems to be expressed at a basic level corresponding to the intracellular-MC present in the cell. Up-regulation in the WT seems to be unnecessary during the first 24 h under this level of HL. Unexpectedly, a basal level of the *mcyE* transcript was observed under control conditions in the MC-deficient mutant that was similar to that in the WT. The disruption of *mcy*D by Cm^R^ has no effect on the expression of *mcyE* gene expression in *P. agardhii*. This absence of any polar effect of the mutation of one *mcy* gene on the others had previously been reported in *Microcystis* [61]. In the MC-deficient mutant, HL induced a significant increase of the *mcyE* transcript that reached its maximum level from 5 to 24 h (Fig. 3.B). The enhancement of the *mcyE* transcript induced by HL conditions in the *ΔmcyD* mutant strain might reflect a requirement for MCs production under such stress. HL is known to cause direct severe damage of the photosynthetic apparatus, and an indirect increase in ROS production (which induces oxidative stress). As a consequence, many different mechanisms and substances may act as cellular defenses in different ways. MCs may be one of them, as suggested by Zilliges and colleagues [19], as MCs bind to cysteine-residues and to specific protein targets involved in photosynthesis processes and against oxidative stress conditions. The possibility cannot be excluded that the depletion or the absence of MCs in deficient cells could increase the damage caused, and may thus contribute to an increase in the susceptibility to environmental stress. This might explain the sporadic changes seen within populations, where MC-producing genotypes can replace non-producing strains in the field under unfavorable conditions [15], [62].

In conclusion, our findings support the hypothesis that MCs have an intracellular function in *Planktothrix agardhii* related to the transcriptional variations of mRNA, and that this could be attributed to the intracellular presence of MCs in the producer cells (related to HL stress). However, further investigations are needed to identify the nature of the interactions between MCs and Hsps modulated-responses (if any), and finally, to define a possible connection between MCs and the primary metabolism of cyanobacteria that produce this “secondary” metabolite.

## Supporting Information

Figure S1 **Examples of melting curve profile of 19 genes investigated in the study.** RG candidates : A – *Rsh*; B – *RpoD*; C – *GltA*; D – *GAPDH*; E – *RpsL*; F –*16S rRNA*; GOIs : G – *ClpC*; H – *HtpG*; I – *GroEL*; K – *GroES*; L – *HspA*; M – *dnaK*; N – *Hsp70 (1)*; O – *Hsp70 (2)*; P – *Hsp70 (3)*; Q – *Hsp 70(4)*; R – *GrpE*; S– *Hsp 40*; T – *HslO*;
(DOC)

Figure S2 **Agarose gel electrophoresis showing specific RT PCR products of the expected size for each gene.** A: Ultra Low Range DNA Ladder (lane 1); *16S rRNA* (246 bp) (lane 2); *Clp* (247 bp) (lane 3); *gltA* (175 bp) (lane 4); *GroES* (168 bp) (lane 5); *GroEL* (205 bp) (lane 6); *DnaK* (191 bp) (lane 7); *HspA* (167 bp) (lane 8); *HslO* (202 bp) (lane 9); *Hsp70*(1) (189 bp) (lane 10); *Hsp70*(2) (183 bp) (lane 11); *Hsp70*(4) (200 bp) (lane 12); *Hsp70*(5) (259 bp) (lane 13); *hsp40* (206 bp) (lane 14); *rpoD* (168 bp) (lane 15); NTC (lane 16) B: Ultra Low Range DNA Ladder (lane 1); *mcyE* (250 bp) (lane 2,3); *GAPDH* (173 bp) (lane 4,5); *GrpE* (181 bp) (lane 6,7); *HK* (186 bp) (lane 8,9) C: Ultra Low Range DNA Ladder (lane 1); *rspL* (152 bp) (lane 2); NTC (lane 3); *rsh* (208 bp) (lane 4)
(TIFF)

Table S1 **Optimal parameters obtained for each transcript and its amplification efficiency.**
(DOC)
