# Supplementary figures and images for: Heat Shock Transcriptional Responses in an MC-Producing Cyanobacterium (Planktothrix agardhii) and Its MC-Deficient Mutant under High Light Conditions
Source: PLoS One. 2013 Sep 4;8(9):e73198. doi: 10.1371/journal.pone.0073198 (PMC3762838; doi:10.1371/journal.pone.0073198)

**Figure S1** :

| A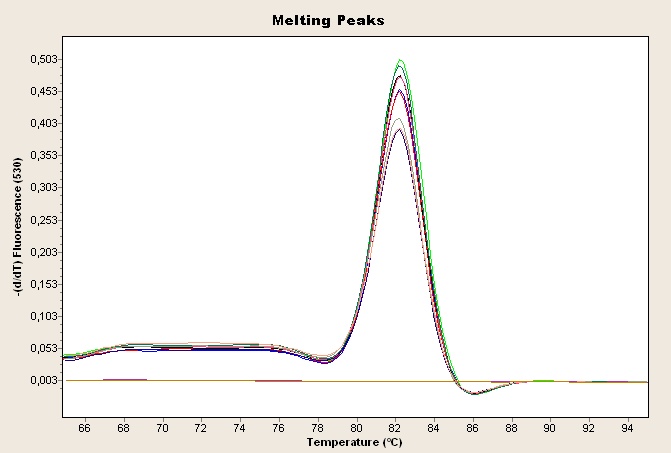 | B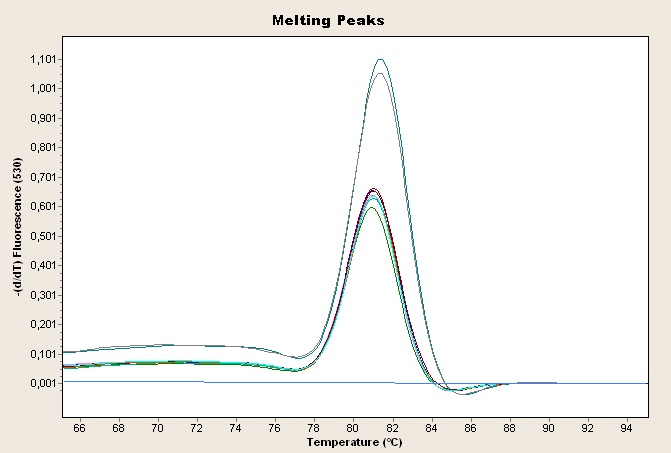 | C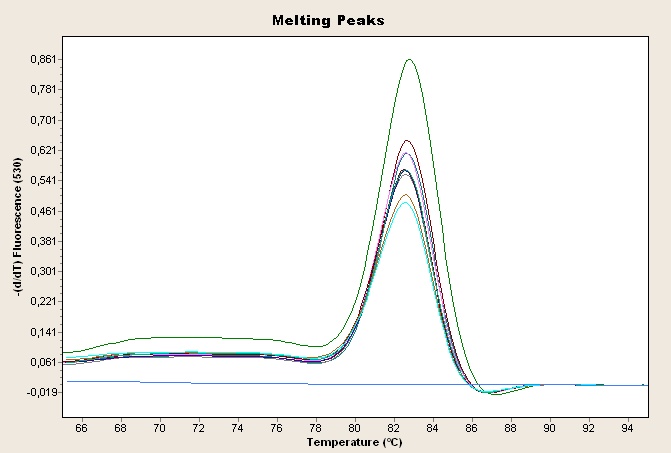 | D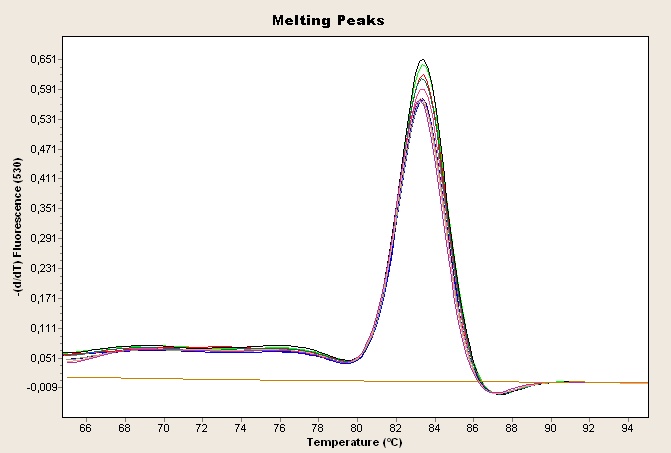 |
| --- | --- | --- | --- |
| E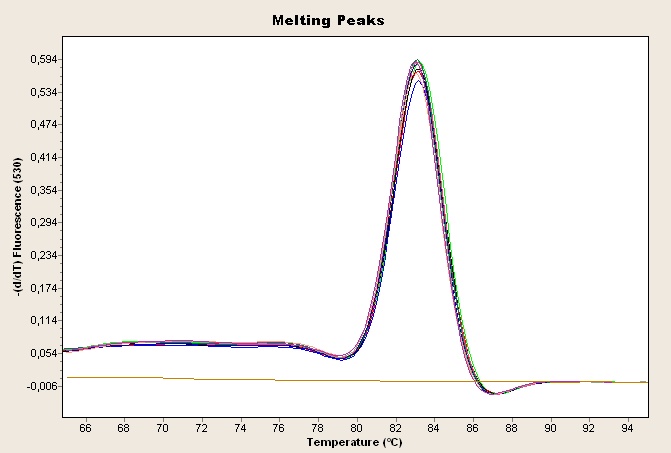 | F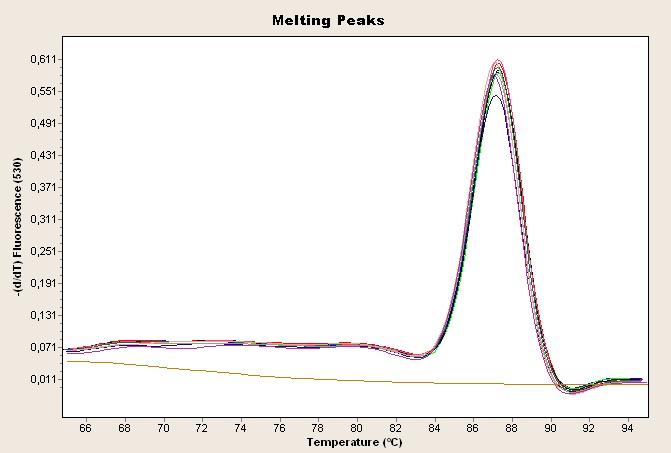 | G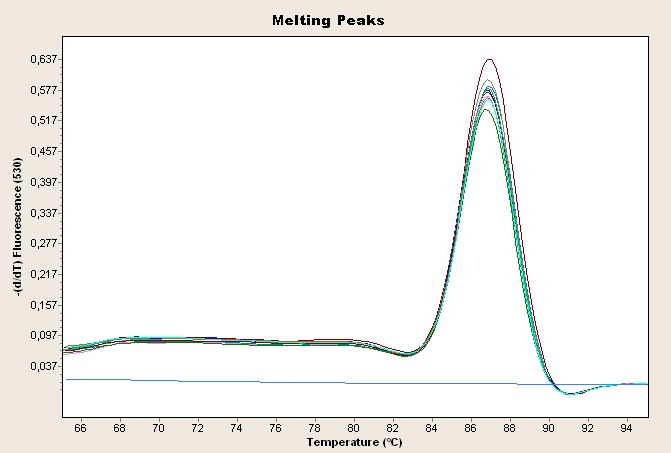 | H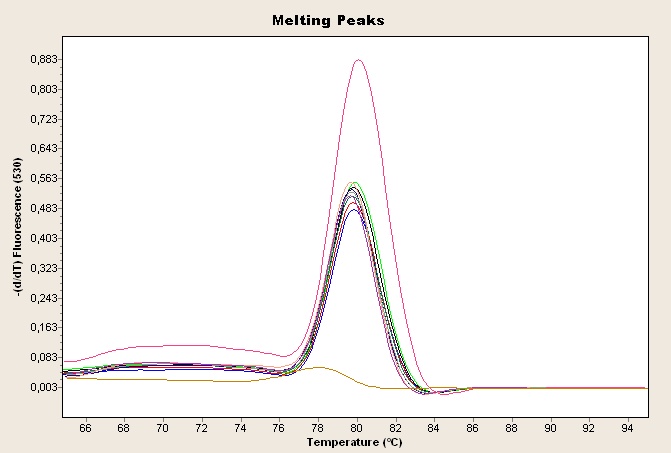 |
| I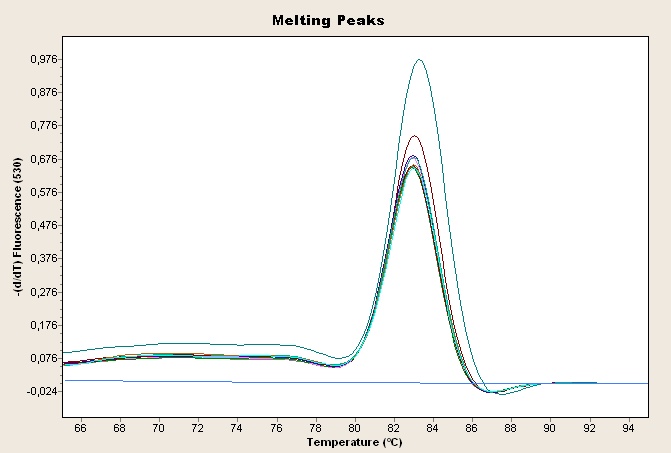 | K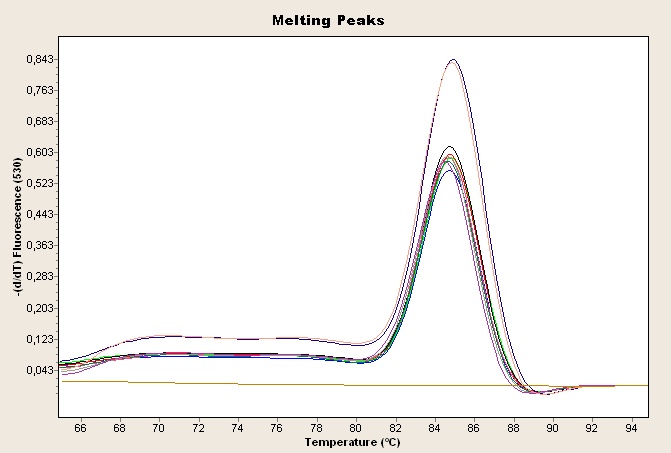 | L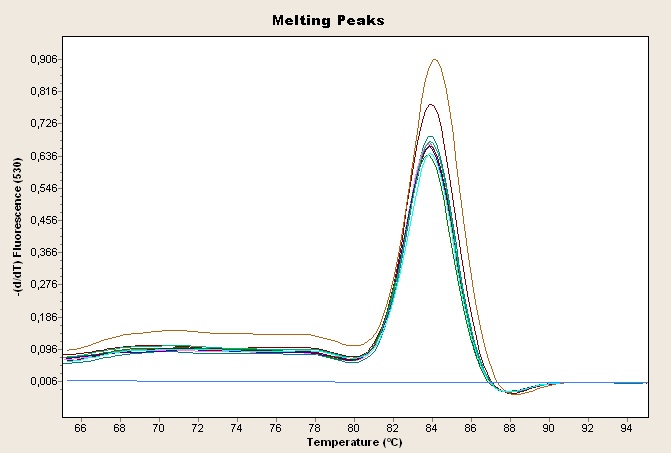 | M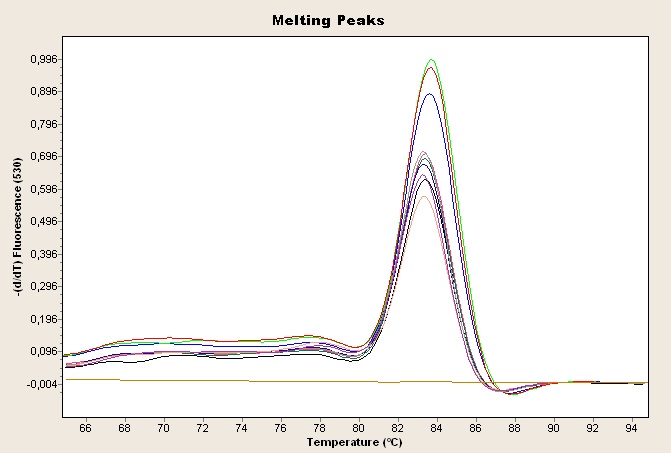 |
| N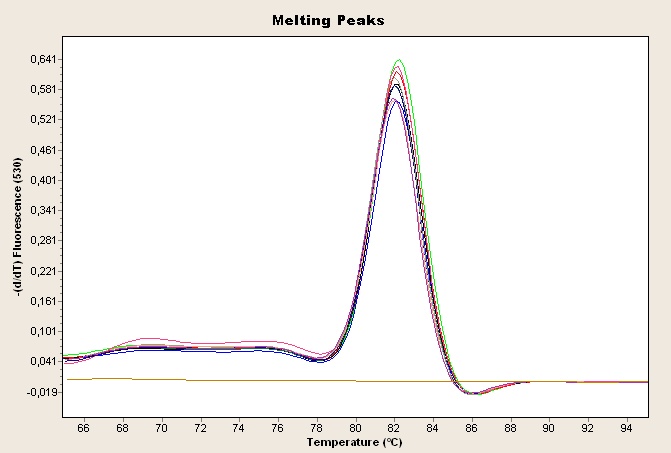 | O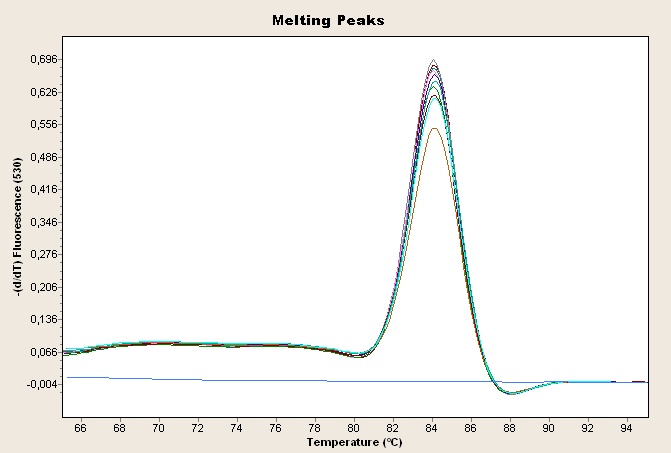 | P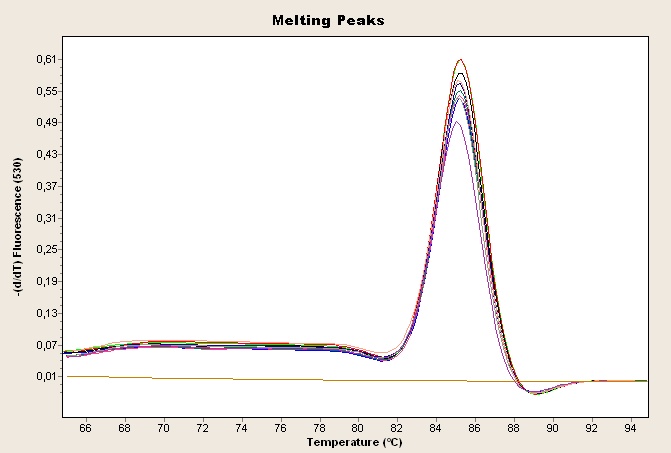 | Q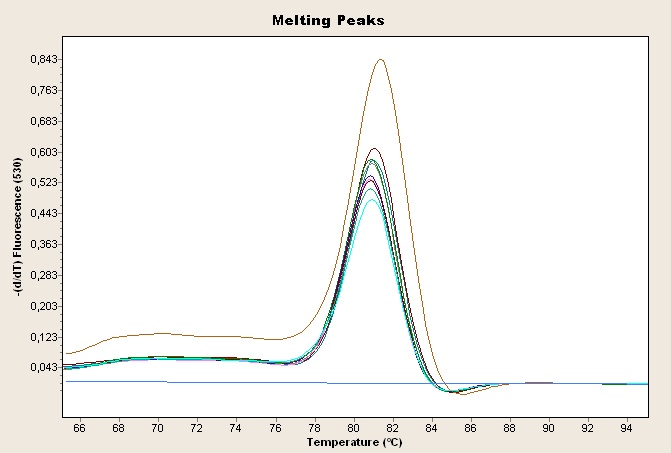 |
| R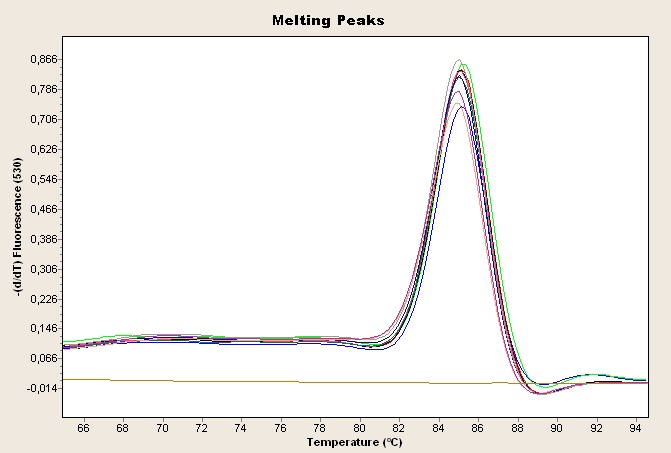 | S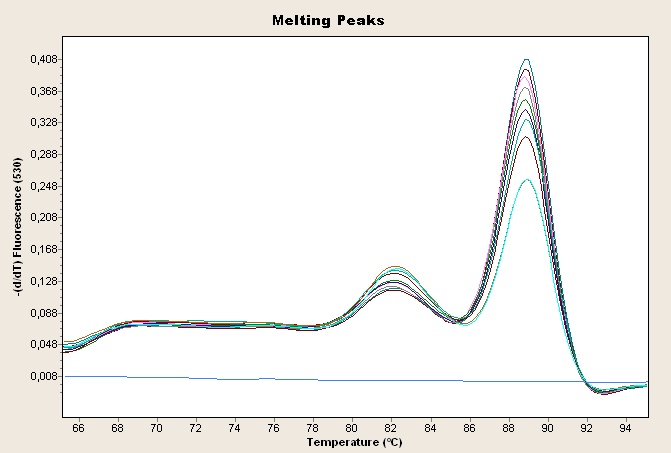 | T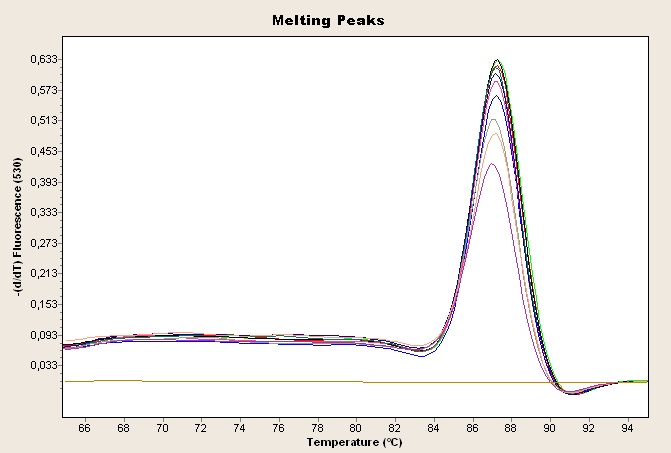 |  |

Supplement: Figure S1 — Examples of melting curve profile of 19 genes investigated in the study. RG candidates : A – Rsh; B – RpoD; C – GltA; D – GAPDH; E – RpsL; F –16S rRNA; GOIs : G – ClpC; H – HtpG; I – GroEL; K – GroES; L – HspA; M – dnaK; N – Hsp70 (1); O – Hsp70 (2); P – Hsp70 (3); Q – Hsp 70(4); R – GrpE; S– Hsp 40; T – HslO; (DOC) [file pone.0073198.s001.doc]
