# Supplementary material for: Heat Shock Transcriptional Responses in an MC-Producing Cyanobacterium (Planktothrix agardhii) and Its MC-Deficient Mutant under High Light Conditions
Source: PLoS One. 2013 Sep 4;8(9):e73198. doi: 10.1371/journal.pone.0073198 (PMC3762838; doi:10.1371/journal.pone.0073198)
Supplement: Table S1 — Optimal parameters obtained for each transcript and its amplification efficiency. (DOC) [file pone.0073198.s003.doc]

**Supplemental materials:**

**Table S1**: Optimal parameters obtained for each transcript and its amplification efficiency

| **Gene** | **Annealing temperature** | **MgCl_2_ concentration (mM)** | **Primer concentration (µM for each)** | **Efficiency** | **R^2^** |
| --- | --- | --- | --- | --- | --- |
| *hspA* | 65°C | 4 | 5 | 0.898 | 0.998 |
| *hslO* | 65°C | 4 | 5 | 0.908 | 0.994 |
| *hsp40* | 65°C | 4 | 5 | 0.918 | 0.999 |
| *grpE* | 62°C | 4 | 10 | 0.869 | 0.998 |
| *dnaK* | 65°C | 4 | 5 | 0.881 | 0.956 |
| *hsp70(1)* | 65°C | 4 | 5 | 0.903 | 0.955 |
| *hsp70(2)* | 65°C | 4 | 5 | 0.967 | 0.984 |
| *hsp70(3)* | 65°C | 4 | 5 | 0.851 | 0.994 |
| *hsp70(4)* | 65°C | 4 | 5 | 0.929 | 0.997 |
| *clpC* | 65°C | 4 | 5 | 0.927 | 0.999 |
| *htpG* | 65°C | 4 | 5 | 0.949 | 0.992 |
| *groEL* | 65°C | 4 | 5 | 0.96 | 0.998 |
| *groES* | 65°C | 3 | 10 | 0.955 | 0.997 |
| *rsh* | 65°C | 4 | 5 | 0.973 | 0.968 |
| *rpoD* | 65°C | 4 | 5 | 0.97 | 0.999 |
| *gltA* | 65°C | 4 | 5 | 0.971 | 0.979 |
| *GAPDH* | 63°C | 4 | 5 | 0.977 | 0.999 |
| *rpsL* | 65°C | 4 | 5 | 0.96 | 0.999 |
| *16S rRNA* | 65°C | 4 | 5 | 0.924 | 0.999 |
